# Dataset on community structure of macro invertebrate fauna in Ubogo river, Udu LGA, Delta State, Nigeria

**DOI:** 10.1016/j.dib.2018.05.084

**Published:** 2018-05-23

**Authors:** S.A. Ejoh, B.A. Unuakpa, T.O.T. Imoobe, S.O. Edeki

**Affiliations:** a Department of Biological Sciences, Covenant University, Ota, Nigeria; b Department of Animal and Environmental Biology, University of Benin, Nigeria; c Department of Mathematics, Covenant University, Ota, Nigeria

**Keywords:** Macro invertebrate, Community structure, Spatial variation physicochemical parameters, Egini and Ubogo Rivers

## Abstract

The datasets contained in this article are based on a baseline study on the selected physicochemical parameters and macro-benthic invertebrates’ community of Egini, and Ubogo Rivers in Delta State for a period of six months: February - July, 2010, within in six stations shared equally among the two rivers using the three communities they flow through as guide and water samples collected on monthly basis from these stations. The objectives include determination of the spatial variations and background concentrations of the selected physicochemical parameters, species composition and abundance of the macro-benthic invertebrates. Sixteen physicochemical parameters were analyzed in the water. Air and water temperature and current velocity were determined in-situ; the rest physicochemical parameters were determined adopting standard methods. Dusting method was adopted in sampling the macro-benthic invertebrates.

**Specifications Table**

**Table:** 

Subject area	*Biological Sciences.*
More specific subject area	*Environmental and aquatic study.*
Type of data	*Tables, and graphs.*
How data was acquired	*Periodic sampling via observational and experimental methods*
Data format	*Analysed.*
Experimental factors	*Pollution index.*
Experimental features	*Community structure and sampling effect.*
Data source location	Ubogo stream, Udu, Lat 5.45׳ – 6.20N, and long 5.24׳ – 6°.20׳E Nigeria.
Data accessibility	*Within this article.*

**Value of the data**

• The dataset- benthic macro invertebrates are sensitive to environmental impacts from both point and non-point sources of pollution.
• The datasets integrate the effects of short-term environmental variations, such as oil spills and intermittent discharges.
• Sampling involved via the dataset is relatively easy and inexpensive.
• The benthic macro invertebrates serve as the primary food source for many species of commercially and recreationally important fishes. Hence, an improvement on the economic and financial status of the dealers within the studied centers [1], [2], [3], [4], [5], [6].
• Benthic macro invertebrates communities can be used to identify sources of impairment.

## 1 Data

**Fig. 1:**
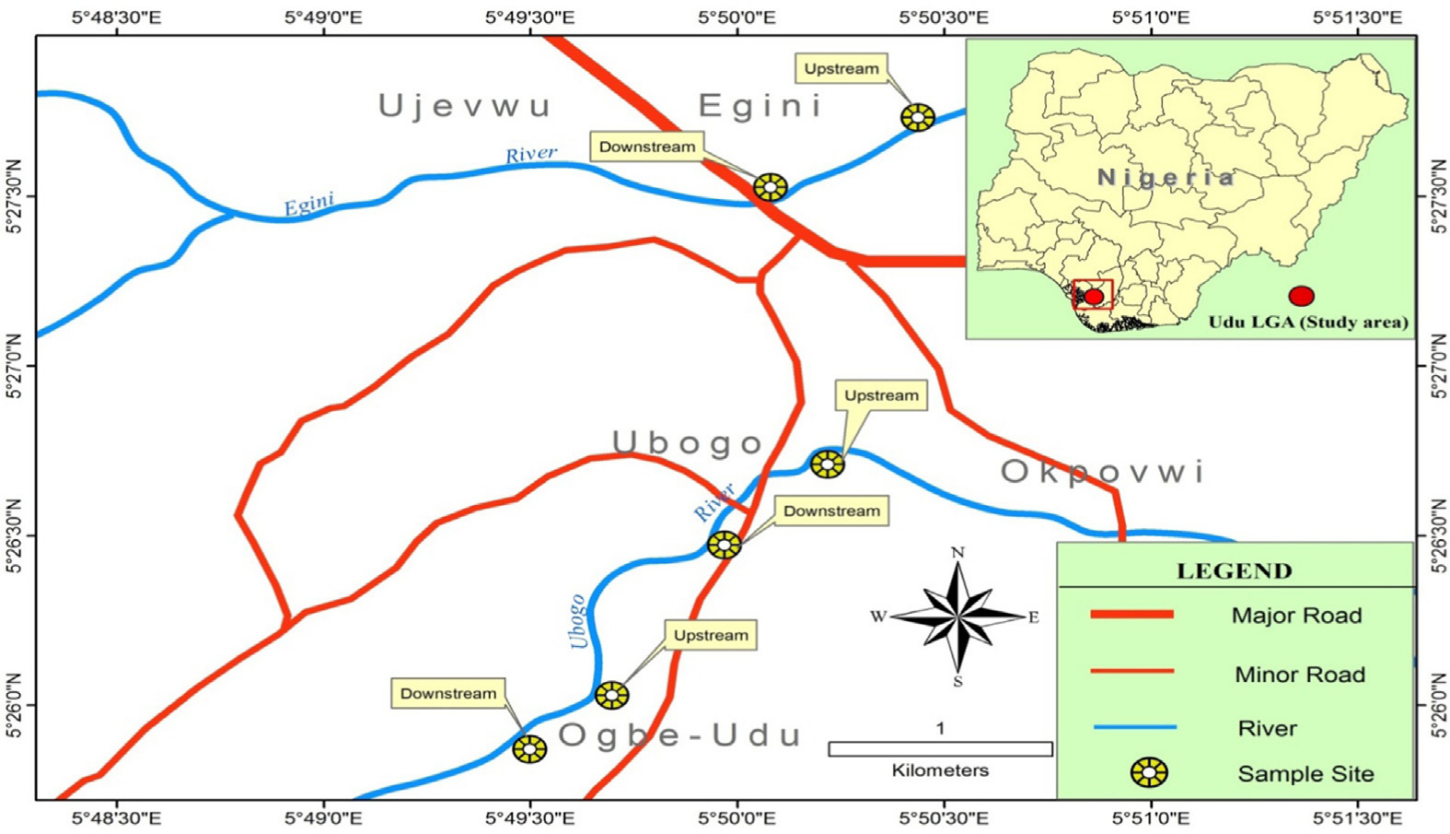
The geology of the Ubogo stream.

**Fig. 2:**
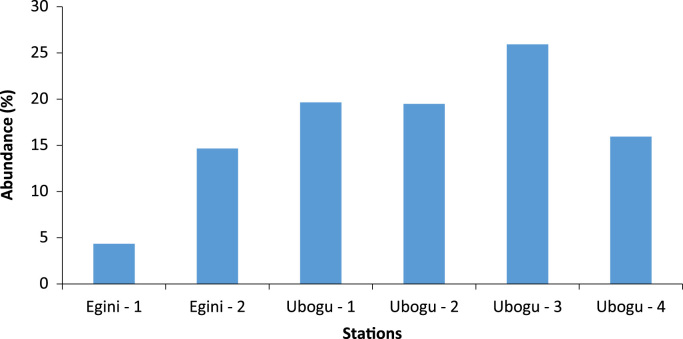
Spatial variations in the overall abundance of the macro-invertebrate fauna at the study rivers.

**Fig. 3:**
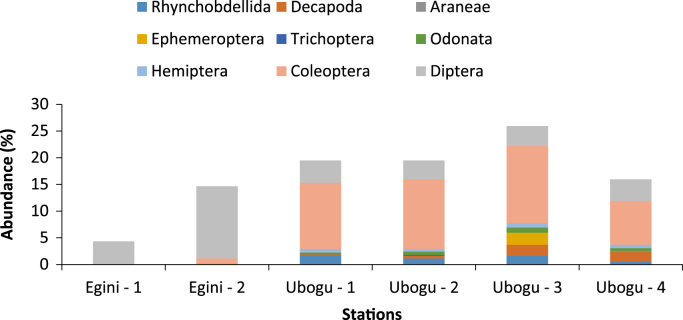
Composition and abundance of the macro-invertebrate fauna at the study rivers.

**Fig. 4:**
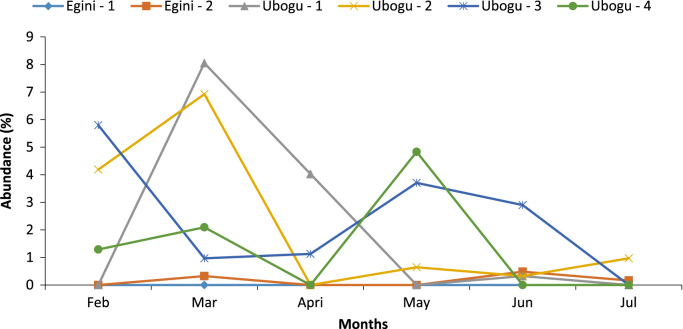
Spatial and temporal variations in the abundance of coleoptera.

**Fig. 5:**
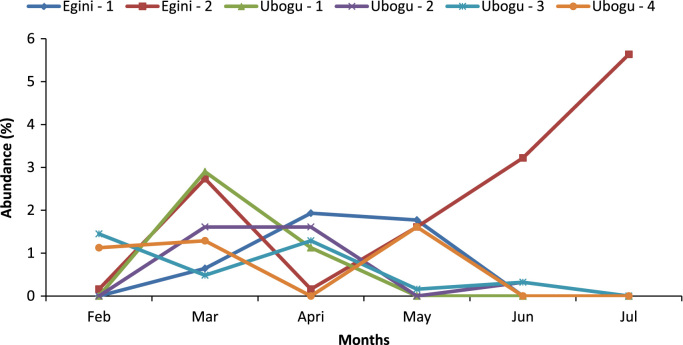
Spatial and temporal variations in the abundance of diptera.

**Fig. 6:**
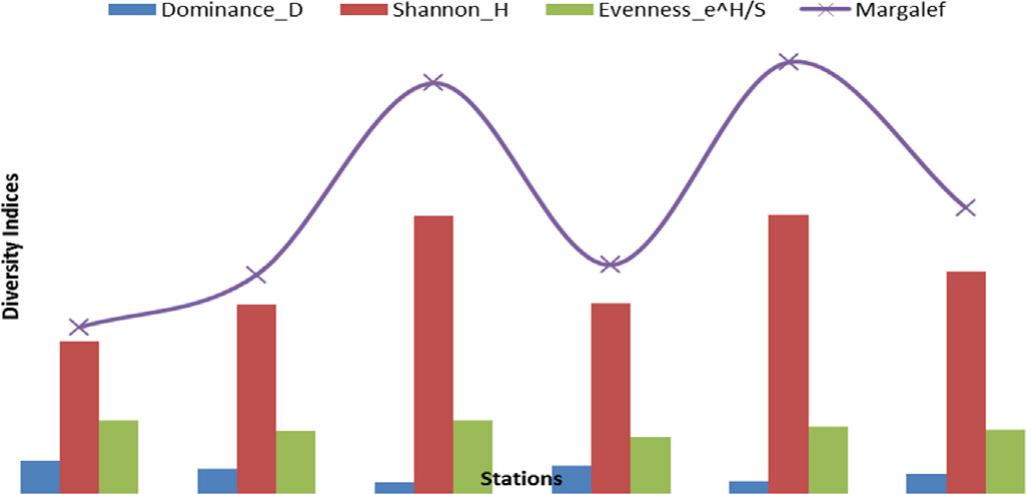
Spatial variations in the various diversity indices. Note: The prevailing conditions as at time samples were collected from these areas.

This study was carried out on Ubogo stream located within latitude (Latitude 5.45׳ – 6.20N, and longitude 5.24׳ – 60.20׳E) with reference to Fig. 1. The streams sourced from Ohworode and flows westerly into Okpare creek at Oto-Udu. Okpare creek empties into Forcados River at Okwagbe-Otor. The streams are about 11 km long. Within the catchment area are located, the Ubogo town, Egini and Ogbe-Udu towns (Fig. 2, Fig. 3, Fig. 4, Fig. 5, Fig. 6).

### 1.1 Sampling locations

In relation to the flow direction, two sampling stations were positioned along Egini River, and four along Ubogo River, two each around Ubogo and Ogbe-Udu community. The sampling stations were visited during the sampling period between February and July, 2010. Sampling stations were chosen on the basis of their proximity to facilities, structure or human activities that could potentially affect water quality and biodiversity. Refs. [7], [8], [9] are referred to related views.

## 2 Experimental design, materials and methods

### 2.1 Methodology and data analysis

#### 2.1.1 Descriptive Statistics and Analysis of Variance (ANOVA)

Correlation analysis was used to determine the relationship between the physico-chemical parameters and the abundance of benthic macro-invertebrate. Non–Parametric Spearman correlation was used.

Basic statistical measurement of central tendency and dispersion was used to characterize stations in terms of physicochemical conditions. Inter station comparisons were carried out to test for significant differences in the physiochemical conditions using parametric analysis of variance (ANOVA). If significant difference (*p*< 0.05) were obtained, Duncan multiple range (DMR) tests were performed to determine the location of differences using the computer SPSS 16.0 window application.

### 2.2 Diversity indices

Diversity indices combine the information on multiple species into a single number. This approach is a common way to summarize data in an environmental study. Data collected at the sampled stations are converted to diversity indices, and then the indices are analyzed to investigate patterns associated with environmental stress.

### 2.3 Community structure

Across the six stations studied in the two rivers, only the bank root biotope was sampled. The macro-invertebrate samples collected from this biotope were analyzed to assess the taxa composition, distribution, abundance, diversity and dominance (Table 1, Table 3, Table 4, Table 5, Table 6).

#### 2.3.1 Composition, distribution, abundance and dominance of macrobenthic invertebrates

The overall taxa composition, abundance and distribution are in Table 2. A total of 41 taxa comprising 621 individuals were obtained. These taxa were encompassed within Rhynchobdellida, Decapoda, Araneae,

**Table 1:** Taxa composition, and relative abundance of Individuals of the macro-invertebrate fauna.

Major taxonomic groups	Percentage of Taxa number	Percentage of relative abundance
Rhynchobdellida	2.44	4.99
Decapoda	9.76	4.67
Araneae	2.44	0.16
Ephemeroptera	7.32	2.42
Trichoptera	2.44	0.16
Odonata	12.20	2.42
Hemiptera	12.20	4.99
Coleoptera	21.95	49.11
Diptera	26.83	33.33
Gastropoda	2.44	0.16

**Table 2:** Relative abundance of the individual MI fauna across the stations at the study areas.

Taxa		Egini-1	Egini-2	Ubogo-1	Ubogo-2	Ubogo-3	Ubogo-4
Rare	Rhynchobdellida	–	–	11	7	10	3
	Decapoda	–	–	1	3	13	12
	Araneae	–	–	–	–	–	1
	Ephemeroptera	–	1	–	–	14	–
	Trichoptera	–	–	–	1	–	–
	Odonata	–	–	2	4	6	3
	Hemiptera	–	–	4	3	5	4
	Gastropoda	–	–	1	–	–	–
Dominant	Coleoptera	–	6	77	81	90	51
	Diptera	27	84	26	22	23	25
	Total	27	91	122	121	161	99

**Table 3:** Summary of the diversity indices across the study area.

	Egini – 1	Egini – 2	Ubogo - 1	Ubogo - 2	Ubogo - 3	Ubogo - 4
Taxa_S	6	10	19	11	21	13
Individuals	27	91	122	121	161	99
Dominance_D	0.30	0.23	0.10	0.25	0.11	0.18
Shannon_H	1.39	1.73	2.54	1.74	2.55	2.03
Simpson_1-D	0.70	0.77	0.90	0.75	0.89	0.82
Evenness_e^H/S	0.67	0.57	0.67	0.52	0.61	0.58
Margalef	1.52	2.00	3.75	2.09	3.94	2.61

**Table 4:** Summary of Bray Curtis Similarity Index.

	Egini - 1	Egini – 2	Ubogo - 1	Ubogo - 2	Ubogo – 3	Ubogo - 4
Egini – 1	1.00					
Egini - 2	0.32*	1.00				
Ubogo - 1	0.19*	0.24*	1.00			
Ubogo - 2	0.08*	0.11*	0.31*	1.00		
Ubogo – 3	0.05*	0.11*	0.43*	0.43*	1.00	
Ubogo - 4	0.08*	0.15*	0.33*	0.66	0.42*	1.00

Degree of relative similarity evaluated from 0=complete dissimilarity to 1=complete similarity; critical level of significance=0.50; Asterisk (*) - indicates significant dissimilarity.

**Table 5:** Summary of Jaccard Similarity Index.

	Egini - 1	Egini – 2	Ubogo - 1	Ubogo - 2	Ubogo – 3	Ubogo – 4
Egini - 1	1.00					
Egini - 2	0.33*	1.00				
Ubogo - 1	0.14*	0.26*	1.00			
Ubogo - 2	0.21*	0.11*	0.25*	1.00		
Ubogo – 3	0.08*	0.15*	0.29*	0.33*	1.00	
Ubogo - 4	0.12*	0.10*	0.19*	0.50	0.36*	1.00

Degree of relative similarity evaluated from 0 = complete dissimilarity to 1 = complete similarity; critical level of significance (C_j_) = 0.5. Asterisk (*) - indicates significant dissimilarity.

**Table 6:** Assessment of the health status of the sampled areas using taxa richness and EPT index.

	Egini - 1	Egini – 2	Ubogo – 1	Ubogo - 2	Ubogo – 3	Ubogo – 4
Taxa Richness Value (Health Status)	6 (Severely impacted)	10 (Severely impacted)	19 (Moderately impacted)	11 (Moderately impacted)	21 (Slightly impacted)	13 (Moderately impacted)
EPT Index Value (Health Status)	0 (Severely impacted)	1 (Severely impacted)	0 (Severely impacted)	0 (Severely impacted)	3 (Moderately impacted)	0 (Severely impacted)

### 2.4 Remarks on physco-chemical conditions

With the exception of turbidity, the rest physico-chemical parameters analyzed in this study were in conformity with Federal Ministry of Environment permissible limits for surface water. Meanwhile the diversity of macro-benthic fauna encountered did not reflect the prevailing physco-chemical conditions encountered. This discrepancy is attributable to the selected biotope sampled, oligotrophic nature of the rivers and also the prevailing turbid condition which in turn can affect the primary production in these ecosystems. It is imperative to characterize the microbial status and heavy metal concentrations of the rivers in order to reach a more concrete conclusion on the health state of the water bodies. Constant monitoring is also advised so that any deviation in the quality of the rivers could be detected on time and appropriate remedial actions taken in time.
